# Philadelphia chromosome duplication as a ring-shaped chromosome

**DOI:** 10.1186/s13039-016-0292-2

**Published:** 2016-11-21

**Authors:** Cesar Borjas-Gutierrez, Juan Ramon Gonzalez-Garcia

**Affiliations:** 1 Division de Genetica. Centro de Investigacion Biomedica de Occidente, Instituto Mexicano del Seguro Social, Sierra Mojada # 800. Colonia Independencia, CP 44340 Guadalajara, Jalisco Mexico; 2 Doctorado en Genetica Humana, CUCS-Universidad de Guadalajara, Guadalajara, Jalisco Mexico

**Keywords:** t(9;22) translocation, Philadelphia chromosome, der(22) chromosome, Secondary chromosomal changes, Duplication, Ring chromosome

## Abstract

The gain of a second copy of the Philadelphia chromosome is one of the main secondary chromosomal changes related to the clonal evolution of cells with t(9;22) in chronic myelogenous leukemia. This gain causes the acquisition of another copy of the *BCR/ABL1* fusion gene. Isochromosomes of the der(22) chromosome or double minute chromosomes are well known to lead an increased copy number of *BCR/ABL1* gene. There is no antecedent of Philadelphia chromosome duplication as a ring chromosome. A recent published report contains evidence that strongly suggests that the Philadelphia chromosome was duplicated as a ring chromosome, observation that was overlooked by the authors. The instability inherent to the ring chromosome increases the risk of emergence of clones containing more and more *BCR/ABL1* gene copies, which would produce increased fitness for clonal selection, resulting in worsening of the patient’s prognosis.

## Letter to Editor

The gain of a second copy of the Philadelphia chromosome (i.e. the der(22) chromosome) is well known to be one of the main secondary chromosomal changes related to the clonal evolution of cells with t(9;22) in chronic myelogenous leukemia [1, 2]. This gain is related to the acquisition of another copy of the *BCR/ABL1* fusion gene. Other rare mechanisms leading to an increased copy number of *BCR/ABL1* gene are either isochromosomes of the der(22) chromosome − with breakpoints located in q10 or in 9q34 bands − or double minute chromosomes [2–5]. No antecedent has been stated of a ring-shaped Philadelphia chromosome duplication [2]. We suspect that the chromosome indicated as der(22) in figure 1 in Wafa et al.’s work [6] is actually a duplicated ring chromosome with an appearance similar to the ring chromosome reported by Ramirez-Dueñas and Gonzalez in figures 2B, 2C, and 3B [7]. Moreover, the der(22) chromosome described in Wafa et al.’s figure 2A [6] is similar to the one showed in figure 2-right bottom in Ramirez-Dueñas and Gonzalez [7]. This chromosome could be a quadruplicated ring chromosome containing four joined copies of the der(22) chromosome, four green 5′-*BCR* signals (instead of the two interpreted as indicated in the figure legend), and four heterochromatic-centromeric regions (figure 2A in [6]). However, the publication of more G-banded and fluorescence in situ hybridization (FISH) images of the chromosome 22 pair is needed in order to fully clarify such interpretations. The origin of duplicated or quadruplicated ring chromosomes is related to the widely known instability of ring chromosomes [7–9]. In this patient, a clone with a single der(22) ring chromosome was not detected, but such a clone could have easily been replaced by selection of clones with more than one copy of the *BCR/ABL1* gene, i.e. clones with the duplicated ring chromosome.

Abbott’s LSI *ABL1* probe is ~ 650 kilo-base (kb) long and covers a continuous region spanning away through the *ABL1* gene in both directions (proximal and distal) (Abbott Molecular/Vysis, Des Plaines, IL, USA); whereas the size of the entire *ABL1* gene sequence is about 53 kb long [UCSC; http://genome.ucsc.edu/(GRCh38/hg38)], and the translocated *ABL1* gene segment is ~ 34 kb long. We hypothesize that most of the sequence covered by the LSI *ABL1* probe was deleted during the ring formation. The *ABL1* translocated sequence was undetected by FISH [6] probably because the emitted fluorescence of the residual labeled probe was not strong enough for microscopic detection. Then, it is reasonable to deduce the existence of the *BCR/ABL1* gene in the ring chromosome from two actual findings: first, the detection of the b3a2 hybrid transcript by reverse transcription-polymerase chain reaction after Imatinib treatment [6]; and second, the visualization of the 5′-*BCR* sequence only on the ring chromosome (figure 2A in [6]).

In considering the lack of specific criteria for describing such kind of rearrangements in the ISCN (2013) [10], the duplicated ring chromosome could be described as der(22)r(22;9)(::22p13→22q11.2::9q34::9q34::22q11.2→22p13::).

On the other hand, the “clone” with 48,XY,+8,+8,t(9;22)(q34;q11.2),inv dup(22) observed in only one cell [6] should not be considered as a clone because it does not fulfill the ISCN (2013) criterion depicted in point 11.1.1 [10]. In other words, this single cell could result from the clone with 49 chromosomes due to an artificial loss of one chromosome 19. In contrast, the authors discussed nothing regarding the gain of one chromosome 22 in the cell depicted in figure 2C [6]. One of these two supposed chromosomes 22 could be either the unobserved single der(22) ring chromosome or a rod-shaped ring similar to that reported by Zuffardi et al. [9].

In conclusion, according to evidence presented by Wafa et al. [6], their report is the first one in literature where the Philadelphia chromosome is duplicated as a ring chromosome. Moreover, the instability inherent to the ring chromosome increases the risk of emergence of clones containing more and more *BCR/ABL1* gene copies, which would produce increased fitness for clonal selection, resulting in worsening of the patient’s prognosis.

## Abbreviations

FISH: Fluorescence in situ hybridization
ISCN: An international system of human cytogenetic nomenclature
Kb: Kilo-base
